# Preoperative periodontal treatment and its effects on postoperative infection in cardiac valve surgery

**DOI:** 10.1002/cre2.212

**Published:** 2019-07-10

**Authors:** Hitomi Suzuki, Koichiro Matsuo, Mieko Okamoto, Haruka Nakata, Hitomi Sakamoto, Mirai Fujita

**Affiliations:** ^1^ Department of Dentistry and Oral‐Maxillofacial Surgery, School of Medicine Fujita Health University Toyoake Japan; ^2^ Doctorate Course for Oral Health and Welfare Niigata University Graduate School of Medical and Dental Sciences Niigata Japan; ^3^ Department of Dentistry and Oral‐Maxillofacial Surgery Fujita Health University Hospital Toyoake Japan

**Keywords:** oral health care, perioperative oral hygienic management, ventilator‐associated pneumonia

## Abstract

**Objectives:**

Oral infection control is important for patients undergoing cardiac valve replacement (CVR) as prophylaxis for postoperative complications. This study examined the changes in oral health status by preoperative periodontal treatment and its effects on postsurgical complications in CVR patients.

**Material and methods:**

We recruited 64 patients undergoing CVR who received preoperative periodontal treatment at our hospital as the intervention group and retrospectively reviewed the medical records of 38 patients who had undergone CVR surgery without dental intervention as the control group. Oral health status was assessed at the first visit to our dental office, 1 day before surgery, and >7 days after surgery. Days of high fever, antibiotics use, and postoperative hospitalization were recorded for the intervention and control groups for statistical comparisons.

**Results:**

In the intervention group, oral health status significantly improved from the initial visit to >7 days after surgery. There were significantly fewer days of high fever (>37.5°C) in the intervention group than in the control group, with comparable results for other events.

**Conclusions:**

This study's findings suggest that preoperative periodontal treatment can improve oral health status surrounding CVR surgery and could be the contributor of the reduction in the risk of postoperative infection.

## 1 INTRODUCTION

As a chronic oral inflammatory disease, periodontitis has strong associations with diabetes, arteriosclerotic disease, rheumatism, infective endocarditis (IE), pneumonia, and other systemic inflammatory conditions (Bagyi et al., 2009; Hajishengallis, 2015; Nishimura et al., 2017; Saengtipbovorn & Taneepanichskul, 2014; Sfyroeras, Roussas, Saleptsis, Argyriou, & Giannoukas, 2012). Bacteremia develops easily in patients with periodontitis with dental treatment, tooth brushing, or even mastication (Nishimura et al., 2017) when periodontal bacteria penetrate into the microvessels of periodontal pockets. Recent studies have demonstrated pathogenic oral bacteria in heart valves and atheromatous plaques (Nakano et al., 2009; Ohki et al., 2012; Oliveira et al., 2015). Oral bacteria may also serve as pathogens for respiratory infection by entering the lower respiratory tract, with the severity of periodontitis being related to the risk of postoperative pneumonia (Bagyi et al., 2009). Thus, patients undergoing heart surgery are at risk of postoperative circulatory and respiratory infections from periodontitis.

Perioperative oral management in patients receiving heart surgery plays an important role in preventing short‐ and long‐term complications derived from pathogenic oral bacteria, such as reducing the chance of complications following cancer or heart procedures (Pedersen, Larsen, & Hakonsen, 2016; Shigeishi et al., 2016). After heart surgery, many patients are subject to extended endotracheal intubation in the intensive care ward due to general condition instability, which increases the risk of ventilator‐associated pneumonia. Prolonged intubation is also a risk factor for dysphagia (Brodsky et al., 2014; Skoretz, Yau, Ivanov, Granton, & Martino, 2014) and subsequent aspiration pneumonia. Therefore, the improvement of periodontal conditions is important to reduce the risk of IE from blood translocation of oral bacteria and postoperative aspiration pneumonia after valve replacement surgery (DeRiso, Ladowski, Dillon, Justice, & Peterson, 1996; Yasny, 2010).

The above studies support that preoperative oral health care can decrease the risk of postoperative complications. However, the precise changes in periodontal condition and oral health status by perioperative professional oral health management remain unknown. The present study examined oral health status changes by preoperative periodontal treatment and its influence on postoperative complications in cardiac valve replacement (CVR) patients.

## 2 STUDY POPULATION AND METHODOLOGY

### 2.1 Participants

In this single‐center cohort study, we prospectively recruited 64 patients who underwent CVR between August 2014 and May 2016 at our hospital as the dental intervention group. We retrospectively reviewed the medical records of 38 patients receiving CVR surgery without dental intervention between April 2012 and July 2014 as the control group. Patients were excluded if they were edentulous, of unstable general physical condition, required reoperation, had IE before surgery, underwent transcatheter aortic valve implantation, or were discharged by death. Information on primary illness, comorbidities, and treatments were extracted from patient records. This study's protocol was approved by the Institutional Review Board of Fujita Health University (Approval ID: 15‐033). All participants in the intervention group provided written informed consent prior to study enrollment.

### 2.2 Data collection

In the intervention group, dental and periodontal examinations were performed at the initial visit to our dental clinic. Periodontal examination was also conducted at 1 day before surgery and >7 days postoperatively. Intensive periodontal treatment was administered to the intervention group before surgery. Dental hygienists performed professional teeth cleaning 1 day preoperatively, and bedside oral care was given at 1 and 4 days after surgery. Immediately postsurgically, oral health care was given at least three times a day by nurses, after which the patients themselves brushed three times a day after recovery of general condition. Oral health instruction by dental hygienists was continued until discharge.

The number of days from the first visit to operation and number of dental treatments at the dental clinic were calculated for the intervention group along with treatment contents. Regarding oral health status, the percentage of teeth with periodontal pockets of 4 mm or deeper, percentage of teeth that were bleeding on probing (BOP) positive, and O′Leary's plaque control record (PCR) were examined at the three time points: the first visit, 1 day before surgery, and >7 days postoperatively.

For both the intervention and control groups, the following postoperative events were extracted from medical charts: days to start of oral feeding, days to discharge, days of endotracheal intubation, days of high fever (≥37.5°C), and days of antibiotics use. Overall comorbidity was evaluated with Charlson comorbidity index (CCI) for both groups.

### 2.3 Data analysis

In the intervention group, the changes in oral health status at the first dental visit, 1 day before surgery, and >7 days after surgery were evaluated using the Friedman test. The Wilcoxon test with Bonferroni correction was adopted for multiple comparisons.

The differences in postoperative events between the intervention and control groups were tested with analysis of covariance with adjustment by the type of surgery and CCI. Statistical analyses were performed using IBM SPSS Statistics 24.0 software (IBM, Armonk, NY, USA). The critical value for rejecting the null hypothesis was *P* < .05.

## 3 RESULTS

### 3.1 Participant characteristics

A total of 102 patients were included in the study and summarized in Table 1. There were 64 patients in the intervention group (39 male and 25 female, mean ± standard deviation [*SD*] age: 69.0 ± 10.3 years) and 38 patients in the control group (25 male and 13 female, mean ± *SD* age: 62.0 ± 19.0 years). There was no remarkable age difference between the groups. All the patients in both groups received a transthoracic cardiac surgery regardless of the surgical procedure. In the intervention group, the mean ± *SD* number of days from the first dental visit to surgery was 39.7 ± 35.2 days. The mean ± *SD* number of preoperative dental visits was 5.0 ± 3.1, which included oral health instruction, basic periodontal treatment, tooth extraction, and denture adjustment.

**Table 1:** Demographic data of participants

Characteristics	Control (N = 38)	Intervention (N = 64)
Mean ± *SD* age (years)	62.0 ± 19.0	69.3 ± 10.3
Male, *N* (%)	25 (65.8)	39 (60.9)
Cardiovascular disease, *N* (%)		
Aortic valve stenosis	19 (50.0)	30 (46.9)
Aortic regurgitation	1 (2.6)	9 (14.1)
Mitral valve stenosis	3 (7.9)	2 (3.1)
Mitral valve regurgitation	14 (36.8)	23 (35.9)
Tricuspid regurgitation	1 (2.6)	0 (0.0)
Surgical procedure, *N* (%)		
Valvuloplasty	15 (39.5)	23 (35.9)
Valve replacement	23 (60.5)	41 (64.1)
Mean ± *SD* days from the first visit to our dental clinic to surgery	—	38.7 ± 35.2
Mean ± *SD* number of treatments before surgery	—	5.0 ± 3.1
Mean ± *SD* number of periodontal treatments before surgery	—	2.5 ± 1.8

Abbreviation: *SD*, standard deviation.

### 3.2 Changes in oral health status during perioperative period

The median perioperative changes in oral health status are shown in Table 2. Probing pocket depth decreased significantly from the first visit (median [interquartile range]: 16.7 [6.0–33.3]%) to the day before surgery (6.7 [2.0–15.1]%), which persisted to >7 days after surgery (6.9 [2.0–16.4]%; both *P* = .001). BOP also became significantly decreased from the first visit (44.0 [24.0–72.7]%) to the day before surgery (22.2 [3.7–41.5]%; *P* = .017) and had further decreased significantly at the study end point (12.9 [0.0–30.8]%; *P* < .001). PCR did not improve remarkably from the first visit (65.0 [44.0–80.5]%) to the day before surgery (58.6 [27.0–67.0]%; *P* = .612) but was significantly decreased at the final measurement (48.1 [28.0–65.0]%; *P* = .012 for the first visit vs. >7 days after surgery).

**Table 2:** Median values of oral health status at study time points

Oral health status	First visit		1 day before surgery		>7 days after surgery		P value
	N	Median (IQR)	N	Median (IQR)	N	Median (IQR)	
Number of teeth	63	24 (16–26)					
PPD >4 mm (%)	63	16.7 (6.0–33.3)	49	6.7 (2.0–15.1)	60	6.9 (2.0–16.4)	<.001^a^^,^ b
BOP (%)	63	44.0 (24.0–72.7)	49	22.2 (3.7–41.5)	59	12.9 (0.0–30.8)	<.001^a^^,^ b ^,^ c
PCR (%)	61	65.0 (44.0–80.5)	47	58.6 (27.0–67.0)	57	48.1 (28.0–65.0)	.013^b^

Abbreviations: BOP, bleeding on probing; IQR, interquartile range; PCR, plaque control record; PPD, probing pocket depth.

a Significant difference between the first visit and 1 day before surgery.

b Significant difference between the first visit and >7 days after surgery.

c Significant difference between 1 day before surgery and >7 days after surgery.

### 3.3 Comparison of postoperative events between the intervention and control groups

General postoperative events in the intervention and control groups are shown in Table 3. The number of days of high fever was significantly lower in the intervention group than in the control group (*P* = .01). The remaining parameters were comparable between the groups. Days to start of oral feeding, days to discharge, and intubation period had significant association with CCI.

**Table 3:** Postoperative general condition in the intervention and control groups

Days of post operative events	Control (N = 38)				Intervention (N = 64)						
	Valve replacement (N = 23)		Valvuloplasty (N = 15)		Valve replacement (N = 41)		Valvuloplasty (N = 23)		P value (ANCOVA)		
	Mean	(SD)	Mean	(SD)	Mean	(SD)	Mean	(SD)	Intervention	Type of surgery	CCI
Days to start of oral feeding	7.3	(15.8)	1.9	(0.8)	2.8	(2.0)	3.7	(4.5)	.16	.23	**.011**
Days to discharge	27.5	(16.1)	21.8	(8.8)	23.3	(11.6)	22.6	(8.3)	.27	.17	**.007**
Intubation period	1.0	(1.7)	0.5	(0.7)	0.6	(1.0)	0.5	(0.8)	.33	.24	**.047**
Days with high fever	4.9	(4.0)	3.5	(1.6)	2.8	(2.0)	3.1	(2.6)	**.01**	.51	.631
Days with antibiotics use	8.4	(8.4)	4.8	(0.9)	5.4	(2.7)	5.5	(3.3)	.08	.13	.093

Abbreviations: ANCOVA, analysis of covariance; CCI, Charlson comorbidity index; *SD*, standard deviation.

Bold *P* values indicate *P* < 0.05.

## 4 DISCUSSION

In the present study, intensive perioperative oral intervention including preoperative periodontal treatment and postoperative oral health care were provided to patients undergoing CVR surgery in the intervention group. As a result, BOP and probing pocket depth became significantly improved prior to and after surgery, with PCR significantly ameliorating postoperatively. Moreover, the number of days with high fever was significantly lower in patients receiving intervention. Preoperative oral management by dental professionals could reduce the risk of postoperative complications in cancer patients (Akutsu et al., 2010; Ishimaru et al., 2018). However, the changes in oral health condition, especially periodontal status, remain unclear. During hospitalization, oral health condition may deteriorate easily from various factors such as critical general condition (Sjogren, 2011; Terezakis, Needleman, Kumar, Moles, & Agudo, 2011), declined activities of daily living, nonoral feeding (Ohno, Heshiki, Kogure, Sumi, & Miura, 2017), and endotracheal intubation (Muramatsu et al., 2019). Diminished oral health condition has been associated with malnutrition and systemic infection in hospitalized patients. Thus, continuous adequate oral health is needed to prevent complications. Our findings suggest that perioperative oral management can significantly improve oral health condition even in the short hospitalization period surrounding surgery.

Oral bacteria are reported pathogens for pneumonia (El‐Solh et al., 2004; Terpenning et al., 2001), and periodontitis severity has been associated with pneumonia mortality (Awano et al., 2008) and postoperative pneumonia onset (Bagyi et al., 2009). Pneumonia is also highly prevalent among postoperative infections in patients undergoing major heart surgery (Bouza et al., 2006; Hortal et al., 2009). Major heart procedures are highly invasive and often require long‐term endotracheal intubation. Intubation itself carries a risk of aspiration pneumonia and may increase the chance of dysphagia after tube removal (Brodsky et al., 2014; Skoretz, Flowers, & Martino, 2010), resulting in possible aspiration pneumonia as well. Maintaining oral health during the perioperative period may be effective to prevent such adverse outcomes. Bergan, Tura, and Lamas (2014) reported that the introduction of an oral health protocol significantly improved oral conditions and decreased the ratio of postoperative pneumonia. In relation to our historical control group, we observed that oral health status became significantly improved prior to CVR surgery and was maintained throughout the postoperative period, with the number of days of high fever being significantly lower in the intervention group. This was consistent with previous reports and strengthened the argument that preoperative oral intervention could decrease the risk of postoperative infection in patients receiving CVR.

For patients at risk of IE, dental checkup and treatments are recommended to prevent odontogenic infection according to the guidelines for IE prevention (Yasny, 2010). Bacteremia can occur not only by invasive dental treatments such as tooth extraction but also by normal life events such as tooth brushing and mastication (Lockhart et al., 2008). With the DNA of periodontal bacteria reported in atherosclerotic plaques and heart valves (El‐Solh et al., 2004; Fernandes et al., 2014; Nakano et al., 2009; Ohki et al., 2012; Ott et al., 2006), maintaining or improving oral health status prior to CVR surgery represents an important prophylaxis for IE and other complications. Based on our results of preoperative intensive periodontal treatment and oral health education, decreasing periodontal inflammation and improving oral health status, such intervention surrounding heart valve surgery, are advised.

### 4.1 Limitations

This study had several limitations. First, the placement of the study controls in the investigation was historical but not parallel. Oral management by dentists and dental hygienists consisting of preoperative periodontal treatment and postoperative bedside oral health care has been routinely conducted for hospitalized patients undergoing surgery in Japan since its introduction into the national health care system in 2012. With several recent reports on the benefits of perioperative oral health and care now being incorporated into the clinical path of heart valve surgery in hospitals, it is ethically and clinically difficult to establish a control group not receiving perioperative oral inspection and treatment. Accordingly, preoperative periodontal treatment was performed for all participants during the intervention period. Placement of the historical control had the limitation that there was no information of the periodontal status and the history of dental treatment prior to the surgery in the control group. We believe the study samples in both the control and intervention groups were collected from similar population who admitted to our hospital from the surrounding area. Although we have no data of oral/periodontal condition of the control patients, the sampling method could indicate that they had similar oral/periodontal condition in both groups at the time of admission to the CVR surgery. The other limitation was the short observation period during patient hospitalization. Although the risk of respiratory complications is low after discharge, the chance of IE is high at class I in the guidelines for IE prevention after implantation of artificial heart valves (Nishimura et al., 2017). Postdischarge follow‐up is required to elucidate the effect of perioperative oral health intervention on long‐term complications in patients receiving CVR surgery.

## 5 CONCLUSION

The present study examined the short‐term changes in oral health status in patients receiving CVR surgery. Preoperative periodontal treatment significantly improved oral health status prior to surgery. Oral health conditions were maintained even after the operation, indicating that perioperative oral health care and education might motivate patient behavior towards better oral and general health. The number of days of fever was significantly lower in the intervention group. Although the study design was limited with the historical comparative study, the results in this study could support that preoperative periodontal treatment could be the contributor of the reduction in the risk of postoperative infection. It is therefore recommended that patients receiving CVR surgery maintain their oral health status to prevent IE in the short and long terms. Perioperative oral health intervention that includes oral health education is warranted to these ends.
